# In Vivo Biocompatibility of an Innovative Elastomer for Heart Assist Devices

**DOI:** 10.3390/polym14051002

**Published:** 2022-03-02

**Authors:** Barbara Zawidlak-Węgrzyńska, Miroslawa El Fray, Karolina Janiczak, Roman Kustosz, Małgorzata Gonsior, Beniamin Oskar Grabarek

**Affiliations:** 1 Foundation of Cardiac Surgery Development, Artificial Heart Laboratory, Wolności 345a, 41-800 Zabrze, Poland; kjaniczak@frk.pl (K.J.); romankustosz@frk.pl (R.K.); mgonsior@devgomed.com (M.G.); 2 Department of Chemistry, Faculty of Medicine in Zabrze, University of Technology, Academy of Silesia in Katowice, 41-800 Zabrze, Poland; 3 Department of Polymer and Biomaterials Science, Faculty of Chemical Technology and Engineering, West Pomeranian University of Technology in Szczecin, Al. Piastów 45, 71-311 Szczecin, Poland; mirfray@zut.edu.pl; 4 DevGoMed Małgorzata Gonsior-Kustosz, Wrzosowa 77a, 44-144 Zabrze, Poland; 5 Department of Histology, Cytophysiology and Embryology, Faculty of Medicine in Zabrze, University of Technology, Academy of Silesia in Katowice, 41-800 Zabrze, Poland; bgrabarek7@gmail.com

**Keywords:** biomaterials, New Zealand white rabbits, inflammatory process, PET, fatty acid, biocompability, wound healing

## Abstract

Cardiac surgical approaches require the development of new materials regardless of the polyurethanes used for pulsatile blood pumps; therefore, an innovative biomaterial, a copolymer of poly(ethylene terephthalate) and dimer fatty acid (dilinoleic acid) modified with D-glucitol, hereafter referred to as PET/DLA, has been developed, showing non-hemolytic and atrombogenic properties and resistance to biodegradation. The aim of this work was to evaluate in vivo inflammatory responses to intramuscular implantation of PET/DLA biomaterials of different compositions (hard to soft segments). Two copolymers containing 70 and 65 wt.% of hard segments, as in poly(ethylene terephthalate) and dilinoleic acid in soft segments modified with D-glucitol, were used for implantation tests to monitor tissue response. Medical grade polyurethanes Bionate II 90A and Bionate II 55 were used as reference materials. After euthanasia of animals (New Zealand White rabbits, n = 49), internal organs and tissues that contacted the material were collected for histopathological examination. The following parameters were determined: peripheral blood count, blood smear with May Grunwald–Giemsa staining, and serum C-reactive protein (CRPP). The healing process observed at the implantation site of the new materials after 12 weeks indicated normal progressive collagenization of the scar, with an indication of the inflammatory–resorptive process. The analysis of the chemical structure of explants 12 weeks after implantation showed good stability of the tested copolymers in contact with living tissues. Overall, the obtained results indicate great potential for PET/DLA in medical applications; however, final verification of its applicability as a structural material in prostheses is needed.

## 1. Introduction

Materials used in the manufacture of implants and other medical devices must fulfill their intended function without causing any negative consequences for the patient. Medical devices or materials must have appropriate properties in terms of how they interact with the host organism (biocompatibility) and resistance to damaging effects of biological agents. For this reason, medical devices are typically subjected to biological evaluation and biocompatibility studies to evaluate the interaction between the device and the patient’s tissue, cells, or body fluids. The main objective of the device biocompatibility assessment is to protect the patient against potential threats that can result from contact with the medical device [1]. Biocompatibility requirements for medical devices and the biomaterials they are made from are set out in the ISO 10993 standard, “Biological evaluation of medical devices”. The selection of appropriate tests depends on the type of medical device or material and its intended use, as well as the nature and duration of contact of the medical device with the patient’s body. According to the standard, the assessment of biological effects that result from exposure of a medical device or material to the human body may include tests such as cytotoxicity, sensitization, intradermal irritation or reactivity, systemic toxicity, chronic toxicity, genotoxicity, and assessment of local reaction after implantation; if the material is to come into contact with blood, its hemocompatibility is also assessed [2].

One of the most important groups of non-degraded polymers in biomedical applications, and in particular for medical devices and medical devices in contact with blood, are poly(urethanes) (PU). These polymers are used for the production of a number of important medical devices, such as pulsed heart assist pumps, e.g., Berlin Heart Excor VAD, mechanical leaf valves [1], or the implantable artificial heart Syncardia [3], Carmat [4]. Good biostability and hemocompatibility are the main factors that determine the use of these materials in devices that contact blood. Despite these advantages, in long-term applications, some of the polyurethanes show a tendency to creep. Minor surface degradations are potential areas for increased adherence of the thrombotic material, and they may be areas colonized by bacteria [5]; therefore, there is a constant search for new biocompatible construction polymers that will meet the set conditions for the production of pulsed blood pumps, and in particular, will be able to work for a long time under cyclical loads [6].

As part of the applied research project (PBS1/A5/2/2012), an original biomaterial (copolymer of polyethylene terephthalate and dimer fatty acid modified with D-glucitol, hereinafter referred to as PET/DLA) was developed in which the potential area of application was Polish heart support systems for adults (ReligaHeart EXT) and children (ReligaHeart PED). Preliminary studies have shown that this material is non-hemolytic and resistant to biodegradation, and its atrombogenic properties are comparable to commercial polyurethane used for the production of pulsed blood pumps [7,8]. In our previous work, we assessed the cytotoxicity and stability of PET/DLA in simulated body fluid (SBF), as well as thrombogenic and hemolytic properties. We demonstrated acceptable biocompatibility of the material against mouse L929 fibroblasts, low thrombogenicity and hemolytic activity, and no negative effect on human erythrocytes. For this reason, the next important stage of research is to confirm the full biocompatibility of the material in terms of application as a heart prosthesis [9]. An important application of the PET/DLA copolymer is its use as a drug delivery system to specific tissues. Cymbaluk-Płoska et al. [10] created porous microcapsules with the above-mentioned material, inside which carboplatin was placed. They concluded that the developed microencapsulation system could be used for long-term, sustained intraperitoneal release of carboplatin in the treatment of ovarian cancer [10].

However, for long-term applications, implantable biomaterials need to demonstrate the lack of inflammatory response in living tissues. Biomaterial implantology has been used, among others, in the development of glucose biosensors, heart valves and pacemakers, and stents and catheters [11]. Any foreign material introduced into the body may cause adverse reactions in the form of acute or chronic inflammation, granulation, or fibrosis. This leads to implant failure, including damage or reduced functionality of the biomedical device [12]. The inflammatory process triggered by implanted biomaterial has been extensively studied over the past several decades. Tissue response to the implanted material reflects not only the properties of the implant, but can also be affected by the procedure [13]. Damage to the connective tissue is a direct factor that induces an inflammatory response. Changes in the permeability of blood vessels are then observed and their extravasation into the surrounding tissues results in the absorption of released proteins onto the implant surface. Then, a cascade of events is initiated that leads to the formation of a temporary fibrin matrix at the implantation site [14]. The biocompatibility of a material is measured by its ability to perform with an appropriate host response in a specific application. In order to assess material biocompatibility, extended in vitro and in vivo experiments are needed following the ISO 10993 standard. Usually, small animal models are used to monitor chemical processes, electrical potential, and a variety of tissues and extracellular substances. A foreign body response is triggered by implanted material can be manifested by a pH decrease in body fluids to about 5.2 within 14 days of implantation, which promotes corrosion/degradation of the implant. Thus, the occurrence of an inflammatory response, e.g., cell death, cell remodeling in the form of osteolysis, fibrosis, or cell proliferation, can be determined from the animal tests, providing insights into the future performance of a material in a human body [11]. Alijotas-Reig et al. [15] described the case of 45 patients with an autoimmune/inflammatory syndrome induced by adjuvants (ASIA) after biomaterial implantation. These authors concluded that the implantation site changes were preceded by a systemic reaction. They noted abnormalities in the biochemical blood tests as well as the biopsy specimens from organs [15]. In this work, the biocompatibility of new elastomers and their potential for clinical practice was performed, and the results were compared to medical-grade polyurethanes [11] currently used in the production of heart prostheses. The performed research is the next step after previous in vitro studies [9]. In order to assess the biocompatibility of new materials in vivo, the induction of inflammatory reactions after intramuscular implantation of the PET/DLA copolymer was monitored. The results of the blood count and blood smear, as well as the concentration of the inflammatory marker CRP in the tested (PET/DLA materials) and control groups confirmed normal healing of the surgical wound. No significant differences in the values of the parameters were noted during the observation. 

The aim of the research described in this study was to evaluate the in vivo inflammatory reaction to intramuscular implantation of PET/DLA biomaterial with different proportions of rigid to flexible segments.

## 2. Materials and Methods

This study was conducted according to the guidelines of the Declaration of Helsinki and was approved by the Institution of the Bioethical Committee operating at the Medical University of Silesia in Katowice, Poland (no. 119/2014). The tests were carried out in accordance with the guidelines of PN EN ISO 10993-6. Polymer samples for in vivo study were produced on a BOY 35E injection molding machine (Dr. Boy GmbH & Co. KG, Neustadt, Germany), as previously described [9].

### 2.1. Biomaterials

A series of poly(aliphatic/aromatic-ester) copolymers with a variable hard to soft segments ratio and modified with D-glucitol has been synthesized as described elsewhere [9,11]. The hard segments (as in poly(ethylene terephthalate; PET) and soft segments containing dimer fatty acid—linoleic acid; DLA was set as 70:30 wt.% and 65:35 wt.%, respectively. Samples were denoted hereafter as PET/DLA 70% and PET/DLA 65%, respectively. The used weight segments ratio allowed the production of polymeric materials of high transparency. The chemical structure of the synthesized materials was verified by nuclear magnetic resonance (NMR) and Fourier transform infrared spectroscopy (FTIR) analysis following procedures described by us previously [9,16].

The reference biomaterials used during implantation were biocompatible polyurethanes Bionate II 90A and Bionate II 55D [12], which are currently used as construction materials for ReligaHeart EXT and REligaHeart PED blood pumps, and which are in the phase of clinical and preclinical trials, respectively [11,14].

Before implantation, the samples in a form of 10 mm of diameter and 0.5 mm thick discs (prepared by a hydraulic hot press, ReMi-Plast PH10T, Czerwonak, Poland) were sterilized by radiation with a 10 MeV electron beam generated in a linear electron accelerator Elektronika 10/10 at a dose of 25 kGy (ICNT Warsaw, Poland). 

### 2.2. Groups of Animals in the Test and Control Groups and the Course of the Implantation Procedure

The experimental animals were New Zealand white rabbits (n = 49), both sexes, weighing not less than 2 kg, with healthy skin intact, and quarantined for at least 20 days. The control group consisted of animals that had not been implanted. In turn, the study group consists of animals in which the biomaterial—PET/DLA 70% or PET/DLA 65% (as the implanted materials) or Bionate II 90A or Bionate II 55D (the reference biomaterial) was used for implantation. The animals were divided into groups according to the length of observation after implantation and according to the type of the implanted biomaterial and the reference biomaterial (Table 1).

Before the procedure, the animals were premedicated under general anesthesia by subcutaneous injection with 0.1 mg Atropinum sulfuricum WZF (Polfa Warszawa, Warszawa, Poland). Then, after 10 min, 3 mg/kg mc ketamine hydrochloride was administered intramuscularly (Pfizer Europe, Pfizer Europe MA EEIG, Ramsgate Road, Sandwich, Kent, CT13 9NJ, UK) with 1% propofol at a dose of 1.0–2.0 mL (Polfa Warszawa, Warszawa, Poland) by injection into the marginal vein of the ear. The implant placement was performed as follows. The skin over the longest dorsal muscle was cut first. Then, in the exposed muscle on both sides of the spine, pockets with dimensions of approximately 10x10 mm, in which a single, sterile implant was inserted, were “blunt” prepared. The tissues were sutured with a continuous suture (Prolene 4-0 subcutaneous tissue (Johnson & Johnson, New Brunswick, NJ 08933, USA), Mersilene 3-0 skin, (Johnson & Johnson, New Brunswick, NJ 08933, USA)) and subcutaneous injection with 4% tolfedic acid solution (ETOQUINOL BIOWET GORZÓW, Gorzów, Poland). The animals were administered intravenous saline immediately after surgery to avoid dehydration and metabolic disturbances during the recovery period from sedation. The animals were then transferred to separate cages and observed for 4 and 12 weeks. In the control group, only the pocket preparation procedure itself was performed, as described above, without implantation. After the observation period was completed and the tests were performed, the animals were humanely euthanized by pharmacology in accordance with the standards. 

### 2.3. Blood Tests

Blood samples (10 mL) from the test and control animals were collected from the marginal vein of the ear in the following time regime:

For animals under 4 weeks of observation, blood was collected before surgery, 2 weeks after surgery, and before euthanasia.

For animals under 12 weeks of observation, blood was collected before surgery, 4 and 8 weeks after surgery, and before euthanasia.

The following parameters were determined: peripheral blood count (2800 BC VET hematology analyzer, Mindray, Shenzhen, China), blood smear with May Grunwald–Giemza staining, and serum C-reactive protein (CRP, RT-190 4C semi-automatic biochemical analyzer; Stamar, Dąbrowa) Górnicza, Poland).

During blood collection, the naked eye also assessed, in macroscopic examination, the postoperative scar appearance and degree of wound healing. 

### 2.4. Histopathological Examination of Tissues and Organs

After the animal was euthanized, the tissue in the immediate vicinity of the wound and the organs—kidneys, heart, liver, spleen, lungs, and lymph node—in the immediate vicinity of the implantation site was secured for histopathological examinations. Tissue fragments were placed in a 4% formaldehyde solution (POL-AURA, Zabrze, Poland) for 24 h. Then, the samples were successively dehydrated in increasing concentrations of ethyl alcohol 80%, 96%, and 99.8% (POL-AURA, Zabrze, Poland). Organs were impregnated with xylene and were embedded in paraffin. Paraffin sections of 4 µm thick were prepared and were subjected to histochemical staining with the use of hematoxylin and eosin in the Masson tricolor reaction.

During euthanasia, the appearance of the implant and of tissues in its immediate vicinity were assessed with the naked eye. The implant itself was collected for further chemical stability tests.

### 2.5. Assessment of the Chemical Stability of the Material after Implantation 

Samples obtained 12 weeks after implantation were analyzed by gel permeation chromatography and Fourier transform IR spectroscopy. The materials were analyzed for changes in their structure or molecular weight compared to the non-implanted material. For the study, PET/DLA 65% implants were taken from rabbits no. 17, 19, and 21; PET/DLA 70%, implants were taken from rabbits no. 6, 9, and 10.

#### 2.5.1. Fourier Transform Infrared Spectroscopy (FTIR) 

The analysis of material samples (flat shapes) was performed using a Nicolet FTIR 6700 spectrometer (Thermo Fisher Scientific, Waltham, MA USA) equipped with a Smart Orbit™ ATR attachment with a diamond crystal. The spectra were recorded in the range of 4000–450 cm^−^^1^ with a resolution of 4 cm^−1^, making 64 scans. OMNIC™ Series Software (Thermo Fisher Scientific, Waltham, MA, USA) was used for spectra analysis. For each fitting, 4 measurements (two on each side) were made to determine the homogeneity of the material. 

#### 2.5.2. Gel Permeation Chromatography (GPC)

The number and average molecular weights and the degree of polydispersity of the above-mentioned samples were determined by gel chromatography on a chromatograph consisting of a Viscotek VE 1122 pump (Malvern Panalytical, Malvern, UK) and a RI SE-61 differential refractometer detector (Shodex, Shodex, Tokyo, Japan). The separation of the samples was carried out on a system of two high-resolution PLgel 5 μm 2xMixed-C polystyrene columns (Polymer Laboratories, Erie, PA 16510, USA). Chloroform with a flow rate of 1 mL/min was used during the mobile phase. The determinations were carried out at 35 °C.

Analytical solutions were prepared in chloroform and poly(vinylidene fluoride-hexafluoro propylene (PVD-HFP). The obtained solutions with a concentration of about 0.3% were filtered through a 0.45 µm filter. All samples dissolved without visible residue.

The calculations of the number and average molecular weights were made based on conventional calibrations that were generated with low polydispersity polystyrene standards. In addition, the degree of polydispersity was given.

#### 2.5.3. Scanning Electron Microscopy (SEM)

The surface morphology of the copolymer samples was investigated using a scanning electron microscope (Quanta 250 FEG, FEI Company, USA) operating at an accelerating voltage of 10 kV in a low vacuum (80 Pa) according to the manufacturer’s recommendation. 

### 2.6. Statistical Analysis

Statistical analysis was performed with the use of the licensed version of the STATISTICA 13PL program (StatSoft, Krakow, Poland) with a statistical significance threshold (*p* < 0.05). In order to compare the values of blood count and CRP parameters within one group throughout the observation period, the non-parametric Friedeman ANOVA test of variance for dependent groups and the post hoc Friedeman ANOVA test were used. Conversely, the U Mann–Whitney test was used to compare the value of a given parameter between the group of test and control animals at a given moment of observation (the non-parametric equivalent of the Student’s *t*-test for independent groups).

## 3. Results

In all cases, intramuscular implantation of the samples was uneventful. During postoperative observations, no changes in the general behavior of the animals were found. All animals showed normal mobility and appetite, and no significant differences in body weight were observed. 

### 3.1. Results of Peripheral Blood Count, Concentration of the C-Reactive Protein of Test, and Control Animals during the Observation Period

The obtained results of the morphology, CRP, and the microscopic images of the peripheral blood throughout the observation period of the animals undergoing the procedure of implantation of the material were normal and fell within the range of reference values, which proves that the postoperative wound healing processes are correct. The results of the laboratory tests performed were correct in the control group of animals as well. This is a confirmation of compliance with the rules of asepsis during the procedure. Statistical analysis showed no statistically significant changes in the concentration of the parameters determined in both groups and between the groups during the observation period (Friedman’s ANOVA test, U Mann–Whitney test, respectively; *p* > 0.05). Detailed results are included in the supplementary materials (Tables S1 and S2). 

### 3.2. Assessment of the Postoperative Scar and the Degree of Wound Healing

**Figure 1:**
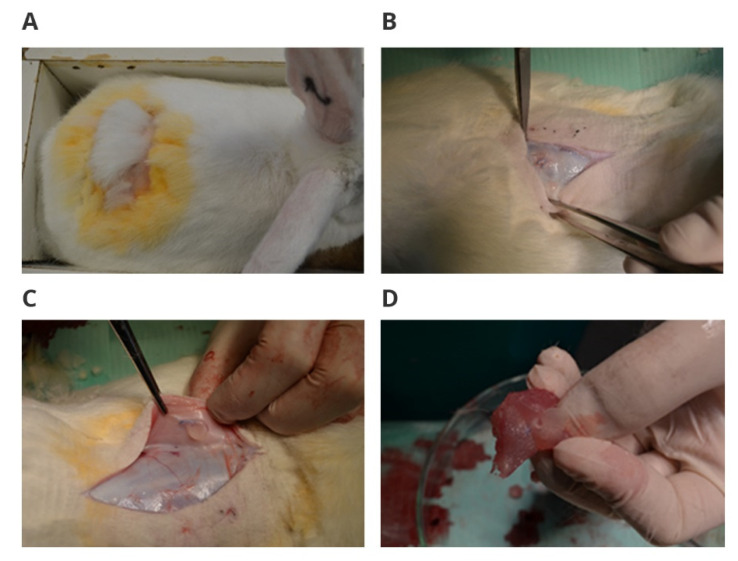
Macroscopically assessed postoperative scar and wound healing after implantation: (**A**) appearance of the postoperative wound 12 weeks after implantation; (**B**) appearance of subcutaneous tissue 12 weeks after implantation; (**C**) appearance of the dorsal subcutaneous tissue 12 weeks after implantation; (**D**) appearance of the longest muscle with an implant 12 weeks after implantation.

Macroscopic examinations analyzing the appearance of the postoperative scar, the degree of wound healing, and the appearance of the tissue around the implant, no pathological changes in organs or at the site of implantation were found (Figure 1).

### 3.3. Results of Histopathological Examination of Tissues and Organs 

Histopathological examinations were carried out in a group of test animals. Tissues were assessed histopathologically in the place where the implant was inserted, along with 3 cm sections of adjacent tissues. The following organs were also collected for biocompatibility assessment: kidneys, heart, liver, spleen, and lungs. Implants were also collected for evaluation.

**Figure 2:**
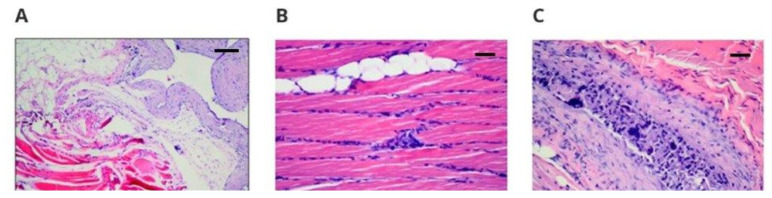
Microscopically assessed image of the site of implantation and the surrounding transverse striated muscles taken from animals with implants, 50× magnification: (**A**) margin, 50× magnification hematoxylin–eosin staining (scale bar 200 µm). Visible fibrotic connective tissue capsule with a slight inflammatory path; (**B**) transverse striated muscle 100% × magnification (scale bar 100 µm), hematoxylin–eosin staining visibly clear and significant inflammation with histiocyte foci and histiophagocytosis; (**C**) margin, 50× magnification (scale bar 100 µm), hematoxylin–eosin staining, visible resorptive reaction containing large dark multinucleated cells inside the connective tissue scar.

Upon histopathological examination of the left margins, the striated muscles did not show signs of inflammation. The response to implantation varied from the absence of any intramuscular changes (two cases—presumably a section taken outside the implant site) to the presence of connective tissue scars (nine cases) that are most likely remnants of connective tissue capsule tissue, to the presence of a connective tissue capsule (40 cases; Figure 2A). The connective tissue capsule and the scar tissue had a variable intensity of lymphohistiocytic infiltration, which did not penetrate between the striated muscle fibers.

Conversely, the striated muscles of the right margin showed signs of inflammation in three cases: a significant amount in one, with features of histiophagocytosis (Figure 2B), and in the other two, inflammation to a slight degree. This one case may raise the suspicion of local toxicity. In one case, a resorptive reaction containing multinuclear cells was observed (Figure 2C), in another case, the presence of eosinophils was observed, and in the third case, inflammatory granulation tissue with numerous blood vessels or calcifications was observed.

**Figure 3:**
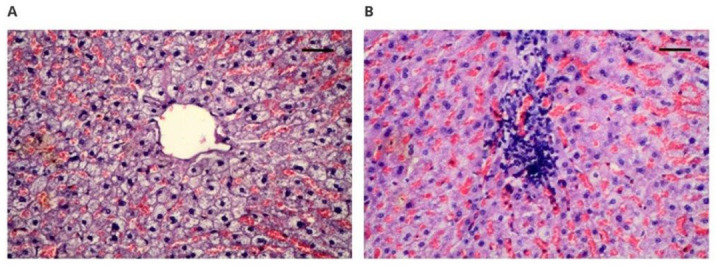
Microscopically assessed image of the heart muscle obtained from implanted animals: (**A**) 100× magnification (scale bar 100 µm), hematoxylin–eosin staining magnification; (**B**) 100× magnification (scale bar 100 µm), Masson’s staining.

Figure 2 shows the microscopic image of the heart in rabbits after 12 weeks of implantation. The cardiac examinations showed that the microscopic image was normal in all examined sections and that no signs of damage to the heart muscle were found. Normal muscle fibers, striation, discrete hyperemia (Figure 3A), and red erythrocytes settling in capillaries were observed, as well as trace fibrosis reflecting the normal presence of connective tissue (Figure 3B).

**Figure 4:**
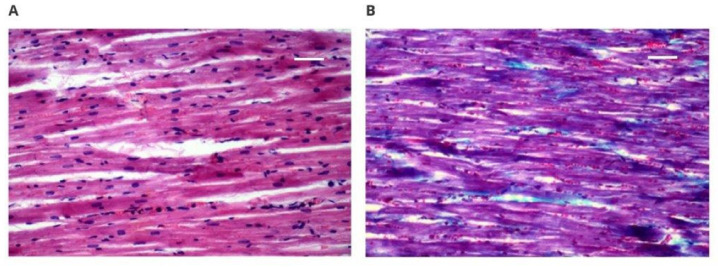
Microscopically assessed image of the liver from the implanted animals: (**A**) 100× magnification (scale bar 100 µm), hematoxylin–eosin staining; (**B**) 100× magnification (scale bar 100 µm), hematoxylin–eosin staining.

Another organ subjected to histopathological examination was the liver. Forty-nine liver specimens were collected for the study. Figure 4 shows a microscopic image of the liver after 12 weeks of implantation. Hepatocytes showed no degenerative, pathological, or toxic changes in the examined cases. In addition, the bile ducts showed no degenerative changes or other pathological changes in the examined cases. A normal system of hepatocytes with a slightly foam-like cytoplasm was also found, which is related to the correct presence of glycogen (Figure 4A). In the interstitium, inflammatory infiltrates were observed in two cases (Figure 4B). In these cases, irrelevant to the tissue localization, there was an abundant infiltration, mainly lymphocytic, with an admixture of histiocytes; however, in view of the absence of changes in hepatocytes, these individual changes should be assigned a marginal significance, presumably due to infection.

**Figure 5:**
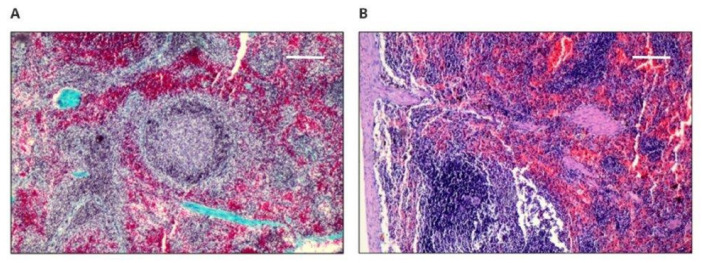
Microscopically assessed image of the spleen collected from animals with implants: (**A**) 50× magnification (scale bar 200 µm), Masson’s staining, white pulp; (**B**) 50× magnification (scale bar 200 µm), Masson’s staining, red pulp.

Figure 5A,B shows a microscopic image of the spleen. Histopathological examinations showed that neither the white pulp nor the red pulp revealed any pathological changes.

**Figure 6:**
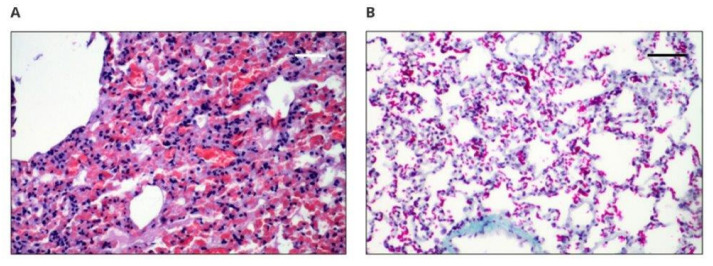
Microscopically assessed image of lungs collected from animals with implants: (**A**) 100× magnification (scale bar 100 µm), hematoxylin–eosin staining; (**B**), 50× magnification (scale bar 200 µm), Masson’s staining.

In the histopathological examinations of the lung specimens, which includes both the right and left lung, the dominant image showed a variable intensity of hyperemia, usually with slight edema; therefore, in order to determine whether the circulatory disorders are acute—related to euthanasia or chronic, long-lasting disorders—observations were made for the presence of siderophages or interstitial fibrosis. There was no presence of siderophages or fibrosis of the alveolar septum, which suggests that the changes resulted from euthanasia (Figure 6B).

**Figure 7:**
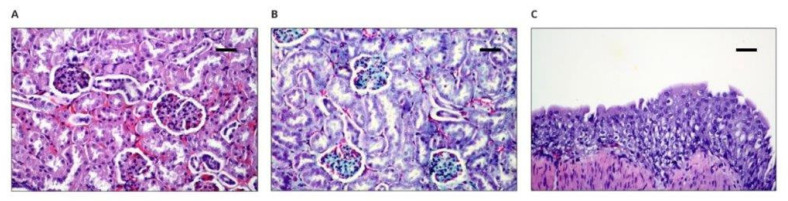
Microscopically assessed image of kidney collected from animals with implants: (**A**) 100× magnification (scale bar 100 µm), hematoxylin–eosin staining, typical coil, and coil structure; (**B**) 50× magnification (scale bar 100 µm), Masson’s staining, typical coil and lobe structure; (**C**) 100× magnification (scale bar 100 µm), hematoxylin–eosin staining, renal pelvic transitional epithelium.

In the histopathological examinations of the kidneys, the renal glows (Figure 7A,B), the urethra, and the pelvis (Figure 7C) did not reveal any pathological changes, including glazing or extensive glomerular fibrosis, the presence of a crescent, intraurethral rollers, or pelvic fibrosis.

### 3.4. The Results of the FTIR and GPC

**Figure 8:**
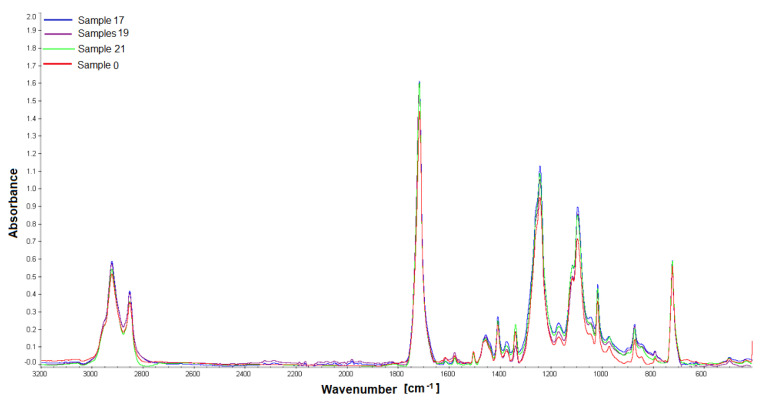
FTIR spectra of the studied PET-DLA 65 copolymers: sample 0-not implanted, sample 17, 19, 21 after 12-week implantation period.

**Figure 9:**
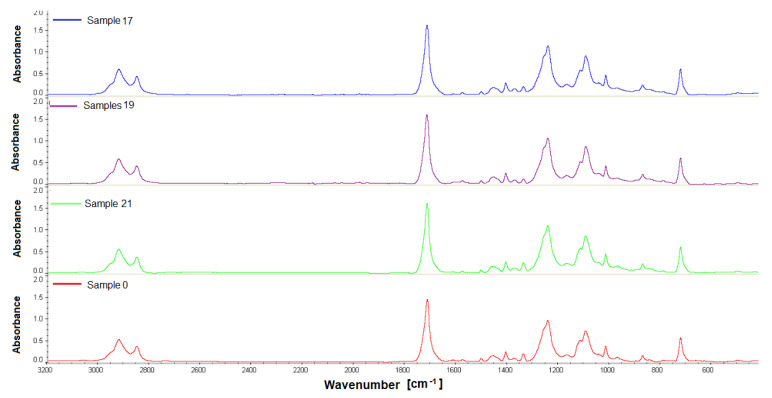
FTIR spectra of the studied PET-DLA 65 copolymers: sample 0-not implanted, sample 17, 19, 21 after 12-week implantation period.

**Figure 10:**
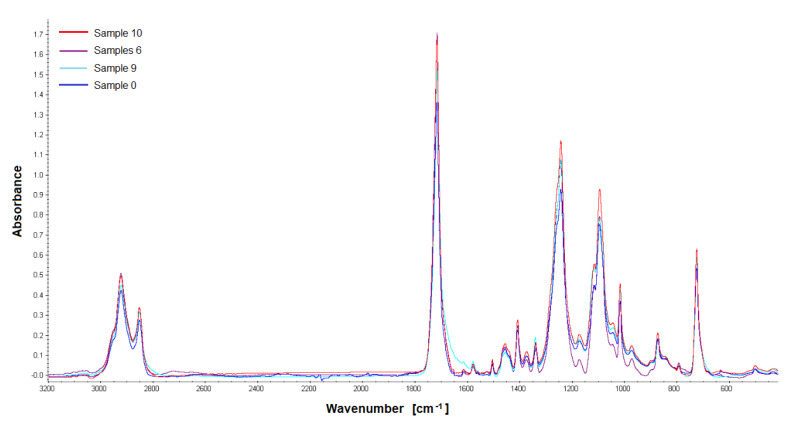
FTIR spectra of the studied PET-DLA 70 copolymers: sample 0-not implanted, sample 6, 9, 10 after 12-week implantation period.

**Figure 11:**
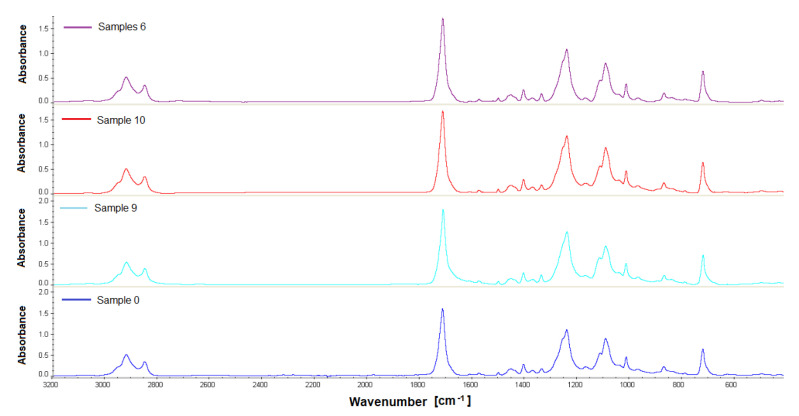
FTIR spectra of the studied PET-DLA 70 copolymers: sample 0-not implanted, sample 6, 9, 10 after 12-week implantation period.

The last stage of our work was the assessment of the chemical stability of the material in the environment of the living tissues of the rabbit body—after intramuscular implantation. FTIR and GPC were used to test the biomaterial samples recovered after implantation. The in vivo impact studies on the polishes were carried out for a 12-week observation period. FTIR analysis showed that the tested material samples were homogeneous; no changes were observed in the FT IR spectra made during four measurements for each sample. The FTIR ATR spectra show characteristic adsorption bands: for materials PET/DLA. Absorption spectrum in the region of 2800–2950 cm^−1^ corresponds to the stretching vibration of the aliphatic and aromatic -C-H. The ester carbonyl bonds can be observed at a wavelength of 1715 cm^−1^. Two peaks at 1240 and 1090 cm^−1^ correspond to the oscillations of the C-O bond stretching in the ester aromatic and aliphatic groups, respectively (Figure 8, Figure 9, Figure 10 and Figure 11). 

The molecular weight characteristics and disparity index of PET/DLA copolymers before implantation and copolymers obtained after euthanasia of rabbits are shown in Table 2. These characteristics for both copolymers PED/DLA 65% and PET/DLA 70% did not change after 12 weeks after injection into the rabbit body.

### 3.5. The Results of the Weight Loss and SEM

In order to demonstrate the good stability of implanted materials, we analyzed the mass of materials before and after implantation and performed SEM analysis (Table 3). The sample weight was evaluated using analytical balance AS 62.R2 PLUS (RADWAG, Radom, Poland).

**Figure 12:**
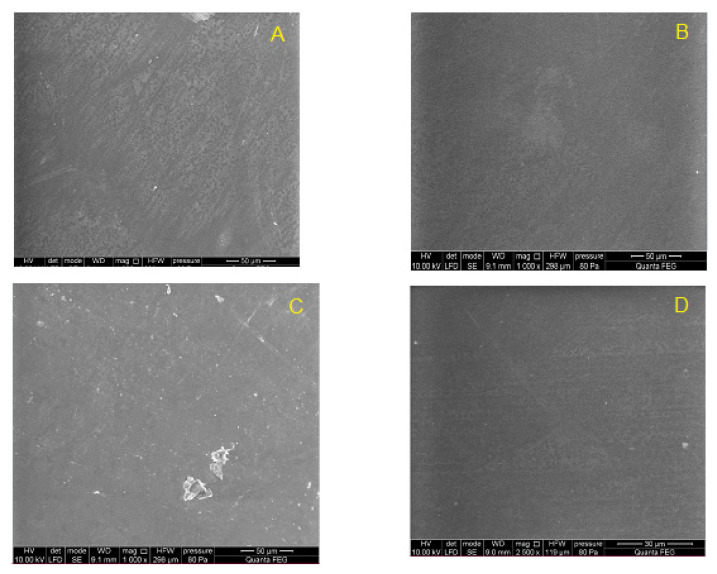
Surface of PET-DLA 65 copolymer: (**A**) not implanted, and (**B**–**D**) after 12-week implantation period.

**Figure 13:**
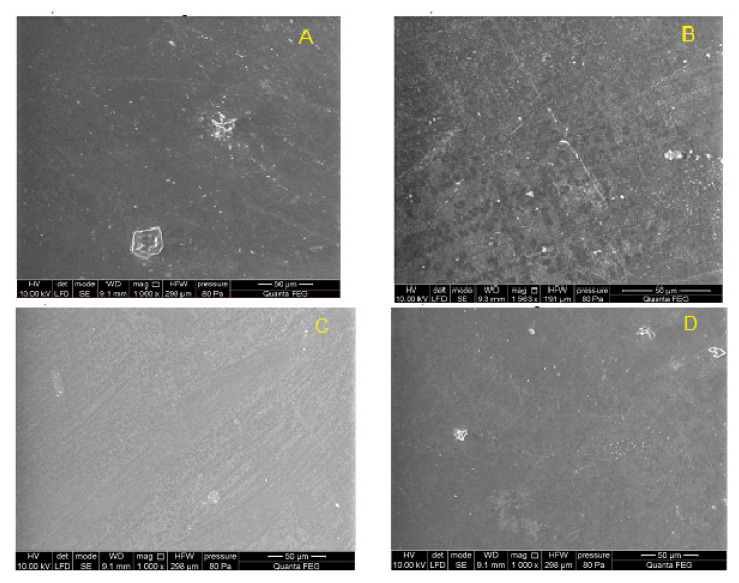
Surface of PET-DLA 70 copolymer: (**A**) not implanted, and (**B**–**D**) after 12-week implantation period.

SEM analysis showed that the surface morphology of the samples after 12 weeks of implantation was smooth, with no evidence of cracks, defects, or degradation damage that may occur in the material during the implantation process. The remaining artefacts reflect the quality of the mold used for samples fabrication by compression molding (Figure 12 and Figure 13).

## 4. Discussion

Gonsior et al. [14] performed the biocompatibility studies of Bionate II 90A and Bionate II 55D biomaterials, which were used as the reference biomaterial in our study. No effect of implanted materials on peripheral blood count parameters and CRP levels were noticed, thus confirming their biocompatibility [14]. Conversely, Berkmortel et al. [17], when using Bionate-55D, Bionate-75D, and Bionate-80A biomaterials, noted that, in the case of hemiarthroplasty materials, the use of materials with significantly lower stiffness than those currently used was requested [17]. This confirms the continuous need to search for new solutions in biomaterial implantology. 

Histological evaluation of organs taken from rabbits after 12 weeks implantation of new materials and control groups was performed. The results of the histopathological evaluation of the examined internal organs for both groups (PET/DLA 70% and PET/DLA 65%), for the control group with medical-grade polyurethane, and for the reference group (without implant) were normal. The liver of most of the examined rabbits showed normal structure. Inflammatory infiltrates were observed in the interstitium of the liver of three animals (rabbits no. 10, 37, and 42). In two cases, without any relation to the tissue localization, non-abundant lymphocytic infiltration with histiocytes was observed. In one case, infiltration was more abundant and was located in the bile duct. Rabbit number 10 was implanted with the tested material, while rabbits 37 and 42 were implanted with the control material, and in light of the lack of changes in the hepatocytes, these single changes should be attributed to a marginal meaning, which was presumably related to the infection. Similar observations were noted in previously performed tests with Bionate II 90A and Bionate II 55D, which did not show any changes in the liver [18]. This observation is the most important because liver damage usually results from the toxic components of polymeric, implanted biomaterial [19]. Markov et al. [20] evaluated the biocompatibility of 1% and 4% hydrogel implanted into Wistar albino rats. Similar to our findings, the researchers reported no significant changes in the histological structure of the liver, kidney, spleen, or lung. Although, between 3 and 8 days after implantation, they noted isolated cases of droplet degeneration of hepatocytes. This was of marginal significance [19]. 

The spleens of the rabbits also showed normal structure. Both white pulp and red pulp did not reveal pathological changes. There was no damage to alveoli, bronchi, or vessels, although inflammatory infiltrates were found in both lungs. Congestion and edema of variable intensity in all specimens (taken for animals with new materials and the control), especially in the central regions, can be considered as the result of euthanasia. The kidneys had a normal histological structure. The results showed that the response to the implanted materials was typical, with the formation of a connective tissue capsule or scar with variable intensity of the inflammatory reaction, which could be the result of mechanical irritation or aseptic inflammation, as no neutrophils were observed [21,22]. Three cases of striated muscle inflammation were observed in rabbits 10, 16, and 22 in the vicinity of implantation with active histiophagocytosis of the muscle and only in the right margin; thus, it was not a systemic process. Moreover, cases of inflammation occurred in rabbits with the implanted new materials (nos. 10 and 16) and with the medical-grade polyurethane (no. 22); thus, it can be concluded that the PET/DLA material did not induce a disorderly reaction during implantation. The observed inflammatory process in the skeletal muscles may be a result of the interaction between the implant and the muscle tissue, as well as a response of the body to the introduction of the biomaterial. Typically, as reported in the literature, the level of cytokine concentration changes as a result of the mentioned interactions, e.g., smooth muscle actin, integrin 1β, and transforming growth factor beta, which in turn causes changes in signaling pathways, resulting in inflammation of the muscle tissue [23,24]. 

Finally, the chemical stability of the new materials after 12 weeks implantation was evaluated by gel permeation chromatography. The results showed no changes in the molecular weight distribution of the polymers before and after implantation. Similarly, no changes in infrared spectra (FTIR) were found; no new signals that could indicate chemical changes in the samples were noticed. 

The factors limiting our observations were the relatively small number of animals in each subgroup and the control of the study. In the perspective of further studies, it would be advisable to consider extending the duration of implantation, as well as extending the panel of biochemical tests by determination of cytokines, growth factors, and immunohistochemistry and histopathological evaluation using other staining techniques. Further analyses are necessary; however, the results obtained in the current study indicate a significant application potential of PET/DLA polymers.

## 5. Conclusions

The obtained results of both biochemical and histological examinations clearly indicate normal healing processes of the postoperative wound for both animal groups (with PET/DLA and control polyurethane). The full histological examination did not show any signs of acute or chronic permanent damage to the organs examined. The healing process observed at the implantation site indicated normal progressive collagenization of the scar, followed by the inflammatory–resorptive process. Chemical tests of the implants after implantation showed good polymer stability in the biological environment in contact with the living tissues of the rabbit organism; however, the new material requires final verification of its applicability as a structural material in prostheses. These studies should be carried out for manufactured prototypes of heart-assisting devices on large animals. 

## Figures and Tables

**Table 1:** Animal groups participating in the study.

Period and Rabbit Number	Implanted Biomaterial	Reference Biomaterial	Implanted Biomaterial	Reference Biomateriał	Control Group
	*PET/DLA 70%*	*Bionate II 55D*	*PET/DLA 65%*	*Bionate II 90A*	*Surgery without Implantation*
**4 weeks**	n = 5	n = 5	n = 5	n = 5	n = 4
**rabbit number**	31–35	42–46	26–30	36–39 and 41	40 and 47–49
**12 weeks**	n = 5	n = 5	n = 5	n = 5	n = 5
**rabbit number**	06–10	21–25	16–21	01–05	11–15

**Table 2:** The molecular weight characteristics and disparity index of PET/DLA copolymers before implantation and copolymers obtained after euthanasia of rabbits.

Copolymer	Duration [Week]	Sample Number	M_n_ [Da]	M_w_ [Da}	Dp
PET-DLA 65	0	0	22,400	48,800	2.3
PET-DLA 65	12	17	22,300	48,600	2.1
PET-DLA 65	12	19	21,400	48,700	2.3
PET-DLA 65	12	21	21,900	48,500	2.2
PET-DLA 70	0	0	22,100	52,900	2.4
PET-DLA 70	12	6	22,200	50,400	2.6
PET-DLA 70	12	9	20,900	48,800	2.5
PET-DLA 70	12	10	21,300	42,200	2.0

Mn, molecular weight; Mw, weight average molecular weight; Dp, Dispersity index; Da, Dalton.

**Table 3:** The weight loss of PET/DLA samples obtained after euthanasia of rabbits.

Copolymer	Duration [Week]	Sample Number	M_o_ [g]	M_i_ [g]
PET-DLA 65	0	0	0.05308	0.05306
PET-DLA 65	12	17	0.05307	0.05302
PET-DLA 65	12	19	0.05627	0.05625
PET-DLA 65	12	21	0.05325	0.05319
PET-DLA 70	0	0	0.05317	0.05316
PET-DLA 70	12	6	0.05060	0.05057
PET-DLA 70	12	9	0.05309	0.05307
PET-DLA 70	12	10	0.05896	0.05893

mo—weight of the samples before implantation process; mi—weight of the samples obtained after euthanasia of rabbits.

## Data Availability

The data used to support the findings of this study is included in the article. The data will not be shared due to third-party rights and commercial confidentiality.

## Supplementary Materials

The following supporting information can be downloaded at: https://www.mdpi.com/article/10.3390/polym14051002/s1. Table S1. The results of peripheral blood counts and CRP during 4—weeks observation of animals in the test and control groups. Table S2. The results of peripheral blood counts and CRP during 12—weeks observation of animals in the test and control groups.
