# Supplementary material for: In Vivo Biocompatibility of an Innovative Elastomer for Heart Assist Devices
Source: Polymers (Basel). 2022 Mar 2;14(5):1002. doi: 10.3390/polym14051002 (PMC8912705; doi:10.3390/polym14051002)
Supplement: Supplementary file 1 [file polymers-14-01002-s001.zip › polymers-1583921-supplementary.pdf]

In vivo biocompatibility of an innovative elastomer for heart assist devices

Barbara Zawidlak-Węgrzyńska [1,2*], Miroslawa El Fray [3], Karolina Janiczak [1], Roman Kustosz [1], Małgorzata Gonsior [1] and Beniamin Oskar Grabarek [4]

Supporting Information

Table S1. The results of peripheral blood counts and CRP during 4 - weeks observation of animals in the test and control groups.

Group	Reference range	Paramater	before implantation			2 weeks after implantation			against euthanasia		
			Me	Q1	Q3	Me	Q1	Q3	M	Q1	Q3
**PED/DLA 65%**	4.10-10.90	WBC (x10^9/L)	7.80	7.10	7.90	7.00	6.80	7.80	6.05	5.66	6.26
	4.20-6.20	RBC (x10^12/L)	6.14	5.99	6.66	6.08	5.75	6.14	128.00	119.00	128.00
	12.00-18.00	HGB (g/L)	133.00	132.00	139.00	126.00	122.00	128.00	41.50	38.30	42.00
	35-51	HCT (%)	42.90	41.50	44.90	40.20	39.60	41.40	229.00	224.00	289.00
	140-440	PLT (x10^9/L)	295.00	251.00	317.00	237.00	221.00	259.00	43.26	42.32	43.59
	< 3.10	CRP (mg/L)	45.77	44.41	46.36	38.85	38.85	40.59	43.26	42.32	43.59
**PED/DLA 70%**	4.10-10.90	WBC (x10^9/L)	9.10	7.40	10.20	7.90	7.50	8.50	8.90	8.20	9.90
	4.20-6.20	RBC (x10^12/L)	6.85	6.75	6.99	5.92	5.89	6.17	6.21	6.09	6.30
	12.00-18.00	HGB (g/L)	145.00	142.00	145.00	129.00	126.00	129.00	132.00	129.00	139.00
	35-51	HCT (%)	46.20	45.80	46.90	40.70	40.10	41.30	42.80	42.00	43.20
	140-440	PLT (x10^9/L)	261.00	228.00	295.00	207.00	185.00	208.00	256.00	246.00	259.00
	< 3.10	CRP (mg/L)	44.80	44.28	46.68	38.56	38.53	38.82	42.86	42.79	44.53
**Bionate II 90A**	4.10-10.90	WBC (x10^9/L)	8.60	8.60	9.50	8.10	6.60	9.30	8.30	6.60	8.40
	4.20-6.20	RBC (x10^12/L)	6.42	6.32	6.85	5.96	5.82	6.00	5.93	5.88	6.09
	12.00-18.00	HGB (g/L)	130.00	127.00	140.00	121.00	120.00	125.00	126.00	125.00	127.00
	35-51	HCT (%)	41.90	40.60	44.30	39.60	38.10	39.90	39.70	39.40	40.50
	140-440	PLT (x10^9/L)	269.00	269.00	286.00	216.00	215.00	238.00	288.00	243.00	325.00
	< 3.10	CRP (mg/L)	48.89	47.66	49.93	38.04	37.98	38.56	42.32	39.04	42.92

**Bionate II 55A**	4.10-10.90	WBC (x10^9/L)	6.70	6.50	8.60	7.60	6.30	7.90	6.70	6.40	7.10
	4.20-6.20	RBC (x10^12/L)	6.28	6.20	6.37	5.79	5.51	6.01	5.75	5.69	6.17
	12.00-18.00	HGB (g/L)	132.00	130.00	138.00	121.00	120.00	125.00	126.00	125.00	126.00
	35-51	HCT (%)	42.00	40.70	43.40	39.00	38.00	39.90	39.60	39.20	39.60
	140-440	PLT (x10^9/L)	214.00	191.00	215.00	205.00	175.00	218.00	246.00	237.00	255.00
	< 3.10	CRP (mg/L)	44.99	44.21	45.58	38.73	38.73	39.02	43.06	42.92	43.12
**Control group**	4.10-10.90	WBC (x10^9/L)	7.85	6.45	8.05	8.60	7.60	9.55	7.20	6.30	7.95
	4.20-6.20	RBC (x10^12/L)	6.38	6.04	6.97	5.94	5.48	6.16	6.00	5.30	6.13
	12.00-18.00	HGB (g/L)	136.00	131.50	143.00	121.50	115.50	123.50	123.50	118.00	128.50
	35-51	HCT (%)	42.30	41.05	44.95	39.60	38.15	39.95	39.45	38.40	40.95
	140-440	PLT (x10^9/L)	313.50	285.00	352.00	263.00	253.00	272.50	343.50	276.00	419.50
	< 3.10	CRP (mg/L)	43.37	41.43	43.89	39.43	38.62	40.19	43.38	43.14	43.43

Me, median, Q1- down quartile; Q3, upper quartile; WBC, white blood cells; RBC, red blood cells; HGB, hemoglobin; HCT, hematocrit; PLT, platelets; CRP, C-reactive protein

Table S2. The results of peripheral blood counts and CRP during 12 - weeks observation of animals in the test and control groups.

Group	Reference range	Paramater	before implantation			4 weeks after implantation			8 weeks after implantation			against euthanasia		
			Me	Q1	Q3	Me	Q1	Q3	Me	Q1	Q3	Me	Q1	Q3
**PED/DLA 65%**	4.10-10.90	WBC (x10^9/L)	8.00	7.80	8.80	7.20	6.60	8.40	4.70	4.10	5.10	8.10	7.70	8.70
	4.20-6.20	RBC (x10^12/L)	6.60	6.16	6.63	6.24	6.01	6.45	5.23	5.06	5.41	6.14	5.95	6.41
	12.00-18.00	HGB (g/L)	142.00	142.00	153.00	129.00	129.00	132.00	116.00	109.00	117.00	140.00	135.00	141.00
	35-51	HCT (%)	44.40	44.20	46.40	43.70	43.60	43.90	37.20	35.50	38.30	44.00	43.30	44.70
	140-440	PLT (x10^9/L)	401.00	358.00	418.00	564.00	479.00	611.00	202.00	181.00	389.00	318.00	257.00	321.00
	< 3.10	CRP (mg/L)	39.83	32.43	41.36	40.96	40.83	41.02	41.21	40.73	41.33	42.26	42.07	42.91
**PED/DLA 70%**	4.10-10.90	WBC (x10^9/L)	8.00	7.70	8.10	8.70	8.60	9.20	8.60	7.80	8.70	8.50	6.60	9.30
	4.20-6.20	RBC (x10^12/L)	7.46	7.17	7.81	6.25	6.11	6.38	6.16	6.03	6.17	5.96	5.81	5.98
	12.00-18.00	HGB (g/L)	150.00	147.00	161.00	140.00	135.00	145.00	128.00	123.00	129.00	129.00	128.00	130.00
	35-51	HCT (%)	52.10	50.60	56.60	43.60	43.20	45.90	42.70	42.60	42.90	41.00	40.80	41.50
	140-440	PLT (x10^9/L)	1146.00	1103.00	1399.00	567.00	567.00	805.00	403.00	282.00	448.00	1148.00	841.00	1453.00
	< 3.10	CRP (mg/L)	45.87	45.79	45.95	48.81	46.75	54.68	41.47	40.58	41.85	39.67	39.21	39.68
**Bionate 55A**	4.10-10.90	WBC (x10^9/L)	9.20	7.90	9.30	7.30	6.20	7.60	5.90	5.40	7.30	9.30	6.70	10.60
	4.20-6.20	RBC (x10^12/L)	6.49	6.39	6.59	5.80	5.62	5.99	5.65	5.23	5.93	5.95	5.45	6.07
	12.00-18.00	HGB (g/L)	148.00	145.00	148.00	127.00	123.00	127.00	123.00	105.00	127.00	132.00	123.00	139.00
	35-51	HCT (%)	45.70	44.00	45.80	41.80	41.00	41.90	41.30	33.90	41.50	42.30	40.40	43.40
	140-440	PLT (x10^9/L)	358.00	298.00	426.00	448.00	257.00	532.00	343.00	147.00	699.00	246.00	205.00	259.00
	< 3.10	CRP (mg/L)	41.29	41.15	42.25	41.15	41.02	41.40	40.91	40.48	41.27	41.42	41.16	42.33
**Bionate 90A**	4.10-10.90	WBC	6.00	5.10	6.80	8.10	7.50	8.10	6.10	3.40	6.20	5.50	5.40	7.10

		(x10^9/L)												
	4.20-6.20	RBC (x10^12/L)	7.42	7.24	7.96	6.58	6.40	7.04	6.14	5.01	6.69	6.24	6.16	6.28
	12.00-18.00	HGB (g/L)	153.00	147.00	154.00	135.00	134.00	152.00	125.00	101.00	135.00	140.00	130.00	140.00
	35-51	HCT (%)	54.30	50.30	54.40	43.80	43.70	50.40	45.30	35.30	47.60	44.60	41.50	44.80
	140-440	PLT (x10^9/L)	742.00	673.00	886.00	546.00	416.00	674.00	399.00	192.00	438.00	528.00	385.00	757.00
	< 3.10	CRP (mg/L)	49.52	49.13	50.59	46.38	45.95	48.11	41.09	40.83	41.15	39.21	38.61	39.21
**Control group**	4.10-10.90	WBC (x10^9/L)	5.30	5.30	6.70	5.20	4.80	6.10	6.10	6.10	7.10	6.70	5.30	9.40
	4.20-6.20	RBC (x10^12/L)	6.35	6.06	6.53	6.32	6.01	6.38	6.13	6.05	6.31	6.06	5.86	6.33
	12.00-18.00	HGB (g/L)	135.00	130.00	140.00	129.00	127.00	134.00	126.00	125.00	133.00	130.00	120.00	146.00
	35-51	HCT (%)	43.00	41.70	45.20	43.30	40.90	43.80	42.20	42.10	43.20	41.70	39.40	46.80
	140-440	PLT (x10^9/L)	696.00	674.00	1294.00	653.00	561.00	889.00	679.00	585.00	1149.00	1294.00	431.00	1804.00
	< 3.10	CRP (mg/L)	40.55	39.94	47.46	45.70	41.15	52.38	40.42	40.30	40.46	40.55	39.94	41.69

Me. median. Q1- down quartile; Q3 . upper quartile; WBC. white blood cells; RBC. red blood cells; HGB. hemoglobin; HCT. hematocrit; PLT. platelets; CRP. C-reactive protein
